# Establishment of embryonic shoot–root axis is involved in auxin and cytokinin response during *Arabidopsis* somatic embryogenesis

**DOI:** 10.3389/fpls.2014.00792

**Published:** 2015-01-14

**Authors:** Ying Hua Su, Yu Bo Liu, Bo Bai, Xian Sheng Zhang

**Affiliations:** State Key Laboratory of Crop Biology, College of Life Sciences, Shandong Agricultural UniversityTaian, China

**Keywords:** shoot–root axis, root apical meristem, cytokinin response, auxin response, somatic embryogenesis, *Arabidopsis*

## Abstract

Auxin and cytokinin signaling participates in regulating a large spectrum of developmental and physiological processes in plants. The shoots and roots of plants have specific and sometimes even contrary responses to these hormones. Recent studies have clearly shown that establishing the spatiotemporal distribution of auxin and cytokinin response signals is central for the control of shoot apical meristem (SAM) induction in cultured tissues. However, little is known about the role of these hormones in root apical meristem (RAM) initiation. Here, we found that the expression patterns of several regulatory genes critical for RAM formation were correlated with the establishment of the embryonic root meristem during somatic embryogenesis in *Arabidopsis*. Interestingly, the early expression of the *WUS-RELATED HOMEOBOX 5* (*WOX5*) and *WUSCHEL* genes was induced and was nearly overlapped within the embryonic callus when somatic embryos (SEs) could not be identified morphologically. Their correct expression was essential for RAM and SAM initiation and embryonic shoot–root axis establishment. Furthermore, we analyzed the auxin and cytokinin response during SE initiation. Notably, cytokinin response signals were detected in specific regions that were correlated with induced *WOX5* expression and subsequent SE formation. Overexpression of the *ARABIDOPSIS* RESPONSE REGULATOR genes *ARR7* and *ARR15* (feedback repressors of cytokinin signaling), disturbed RAM initiation and SE induction. These results provide new information on auxin and cytokinin-regulated apical–basal polarity formation of shoot–root axis during somatic embryogenesis.

## INTRODUCTION

The most critical event during embryogenesis appears to be the formation of the shoot apical meristem (SAM) and root apical meristem (RAM), from which almost the entire plant is post-embryonically established (Meinke, 1991; Scheres, 2007). In the SAM of *Arabidopsis*, *WUSCHEL* (*WUS*) is a critical regulator, and encodes a homeodomain protein that is required for stem cell formation and maintenance (Laux et al., 1996). *WUS* is switched on in the four inner cells of the pro-embryo at the 16-cell globular stage, and is an early molecular marker for SAM initiation in the embryo (Weigel and Jürgens, 2002).

Root growth and development are sustained by the RAM, which is formed during embryogenesis (Sabatini et al., 2003; Petricka et al., 2012). Embryonic RAM formation is initiated at the globular stage, when the uppermost cell of the suspensor—the hypophysis—is recruited in the embryo proper. After asymmetrical division of the hypophysis, the small descendant cell gives rise to the quiescent center (QC), which maintains stem cell identity in the surrounding cells of the RAM to produce a set of differentiated tissues (Möller and Weijers, 2009; Peris et al., 2010). Mutants that fail to form the hypophysis often produce rootless seedlings (Möller and Weijers, 2009). An element required in the QC to maintain columella stem cells is WUS-RELATED HOMEOBOX 5 (WOX5), a putative homeodomain transcription factor (Haecker et al., 2004). In the QC, WOX5 acts in a similar way to WUS in the organizing center (OC) of the SAM, highlighting molecular and developmental similarities between the stem cell niches of both root and shoot meristems (Perilli et al., 2012). In addition, several other putative transcription factors have been shown to contribute to embryonic RAM formation. *PLETHORA* (*PLT*) genes, which belong to the AP2-type transcription factor family, play a key role in the specification and maintenance of root stem cells from early embryogenesis onward (Aida et al., 2004; Galinha et al., 2007). Ectopic *PLT* expression in the embryo induces transformation of apical domain cells into root stem cells (Aida et al., 2004). The SCARECROW (SCR)/SHORTROOT (SHR) transcription factors are required to maintain stem cell activity within the RAM (Di Laurenzio et al., 1996; Helariutta et al., 2000; Sabatini et al., 2003).

Auxin and cytokinin are required for cell differentiation and specification during embryogenesis (Müller and Sheen, 2008; Möller and Weijers, 2009). Asymmetric distribution of auxin mediated by auxin polar transport establishes the apical–basal axis of the embryo, showing that auxin is required for pattern formation of the embryo (Friml et al., 2003; Möller and Weijers, 2009). Cytokinin signaling components function in the hypophysis at the early globular stage of the embryo (Müller and Sheen, 2008). After the first division of the hypophysis, the apical daughter cell maintains the phosphorelay activity of cytokinin signaling, whereas cytokinin signaling is repressed in the basal daughter cell. In early embryogenesis, auxin antagonizes cytokinin signaling through direct transcriptional activation of *ARABIDOPSIS RESPONSE REGULATOR* (*ARR*)*7* and *ARR15*, feedback repressors of cytokinin signaling in the basal cell (Hwang and Sheen, 2001; Müller and Sheen, 2008; Buechel et al., 2010).

Somatic embryogenesis is generally believed to be mediated by a signaling cascade triggered by exogenous auxin (Skoog and Miller, 1957; Sugiyama, 1999, 2000). Indeed, our previous work has shown that the establishment of auxin gradients is correlated with induced *WUS* expression and subsequent embryonic SAM formation during somatic embryogenesis (Su et al., 2009). It has also been suggested that the establishment of the RAM in somatic embryos requires an appropriate auxin gradient (Bassuner et al., 2007). However, the mechanism by which root stem cell specification occurs during early somatic embryogenesis is far from understood. Here, we analyzed the expression patterns of a few critical marker genes involved in RAM formation and development during somatic embryogenesis. Besides the auxin gradients that are established in specific regions of embryonic callus, we found that the spatiotemporal cytokinin response was correlated with RAM formation. Such cytokinin response patterns were critical for spatial induction of RAM-specific genes, such as *WOX5* and *PLT*, and subsequent RAM establishment in the embryonic callus. Our results reveal the distinct functions of cytokinin and auxin signaling required for RAM and SAM induction and shoot–root axis establishment during early somatic embryogenesis.

## MATERIALS AND METHODS

### PLANT MATERIALS

All *Arabidopsis* mutants and transgenic lines used in this study were Columbia ecotypes. The *pWOX5::GFP*, *pPLT2::RFP*, *pSCR::GFP* reporter lines and *plt2*-*1* mutants were kindly provided by Dr. C. Li (Institute of Genetics and Developmental Biology, Chinese Academy of Sciences). *pWUS::DsRED-N7* and *DR5rev:3XVENUS-N7* seeds were kindly provided by Dr E. M. Meyerowitz (Division of Biology, California Institute of Technology, Pasadena, CA, USA). The *ahk2 ahk4* and *ahk3 ahk4* mutants were obtained from Dr. C. Ueguchi (Bioscience and Biotechnology Center, Nagoya University, Nagoya, Japan). The *DR5rev::GFP* lines were provided by Dr J. Friml (Zentrum für Molekularbiologie der Pflanzen, Universität Tübingen, Germany). *pARR7::GFP* and *pARR15::GFP* seeds were provided by Dr J. Sheen (Harvard Medical School, USA). Double reporter lines were generated as follows: *pWOX5::GFP* lines were crossed with *pWUS::DsRED-N7* lines; *DR5rev:3XVENUS-N7* lines were crossed with *pWOX5::GFP* lines, and *pPLT2::RFP* lines were crossed with *DR5rev::GFP* lines; *pARR7::GFP* lines were crossed with *pWUS::DsRED-N7* lines.

### ANTISENSE *WOX5* cDNA PLASMID CONSTRUCTION

To determine the role of *WOX5* during somatic embryo induction, a 754 bp cDNA fragment of the *WOX5* coding region was amplified using the primers 5′-ATACTAGTAAACAGTTGAGGACTTTACATC-3′ (forward) and 5′-ATCTCGAGTACGCATTCCATAACATAGATT-3′ (reverse). The *WOX5* antisense cDNA was then cloned into the estradiol inducible XVE binary vector (Zuo et al., 2000), and transformed into *Arabidopsis* plants.

### GROWTH CONDITIONS, SE INDUCTION AND CHEMICAL TREATMENTS

The growth conditions for *Arabidopsis* plants and somatic embryo (SE) induction followed Su et al. (2009). To induce the transcription of inserted *WOX5* antisense cDNAs, the primary somatic embryos (PSEs) were cultured in embryonic callus-inducing medium (ECIM) with 10 μM estradiol (prepared in DMSO as 10 mM stock; Sigma) for 14 days. Then, the cultured tissues were transferred to somatic embryo-inducing medium (SEIM) with 10 μM estradiol for another 8 days. Estradiol was added every 2 days. The embryonic calli in SEIM were collected for phenotype observation.

### *IN SITU* HYBRIDIZATION

Embryonic calli were fixed in FAA (10% formaldehyde: 5% acetic acid: 50% alcohol) overnight at 4°C. After dehydration, the fixed callus tissues were embedded in paraffin (Sigma) and sectioned at 8 μm. Antisense and sense *WOX5* probes were used for hybridization as previously described by Zhao et al. (2006). The primers used to amplify the 440-bp *WOX5* probes were 5′-CATCATCATCAACCATCAACT-3′ (forward) and 5′-CCATAACATAGATTCTTATATC-3′ (reverse).

### IMAGING CONDITIONS

Somatic embryo morphology was photographed using an Olympus JM dissecting microscope. To detect the fluorescence signals of the marker lines, a Zeiss 510 Meta laser scanning confocal microscope with a 20 × air objective and a 40 × oil-immersion lens was used. Specific sets of filters were selected as described previously by Heisler et al. (2005). The Zeiss LSM software was used to analyze the confocal images. At least 80 samples of each marker line were imaged to confirm the expression patterns at each stage.

## RESULTS

### GENES FOR RAM SPECIFICATION ARE INDUCED DURING EARLY SOMATIC EMBRYOGENESIS

Previously, we described a highly reproducible somatic embryogenesis system in *Arabidopsis* in detail (Su et al., 2009). Green PSEs can be generated from explants (immature zygotic embryos), and then disk-like embryonic calli are produced from PSEs in ECIM containing 2,4-dichlorophenoxyacetic acid (2,4-D). After the calli are transferred to 2,4-D-free SEIM, secondary somatic embryos (SSEs) are induced.

**FIGURE 1:**
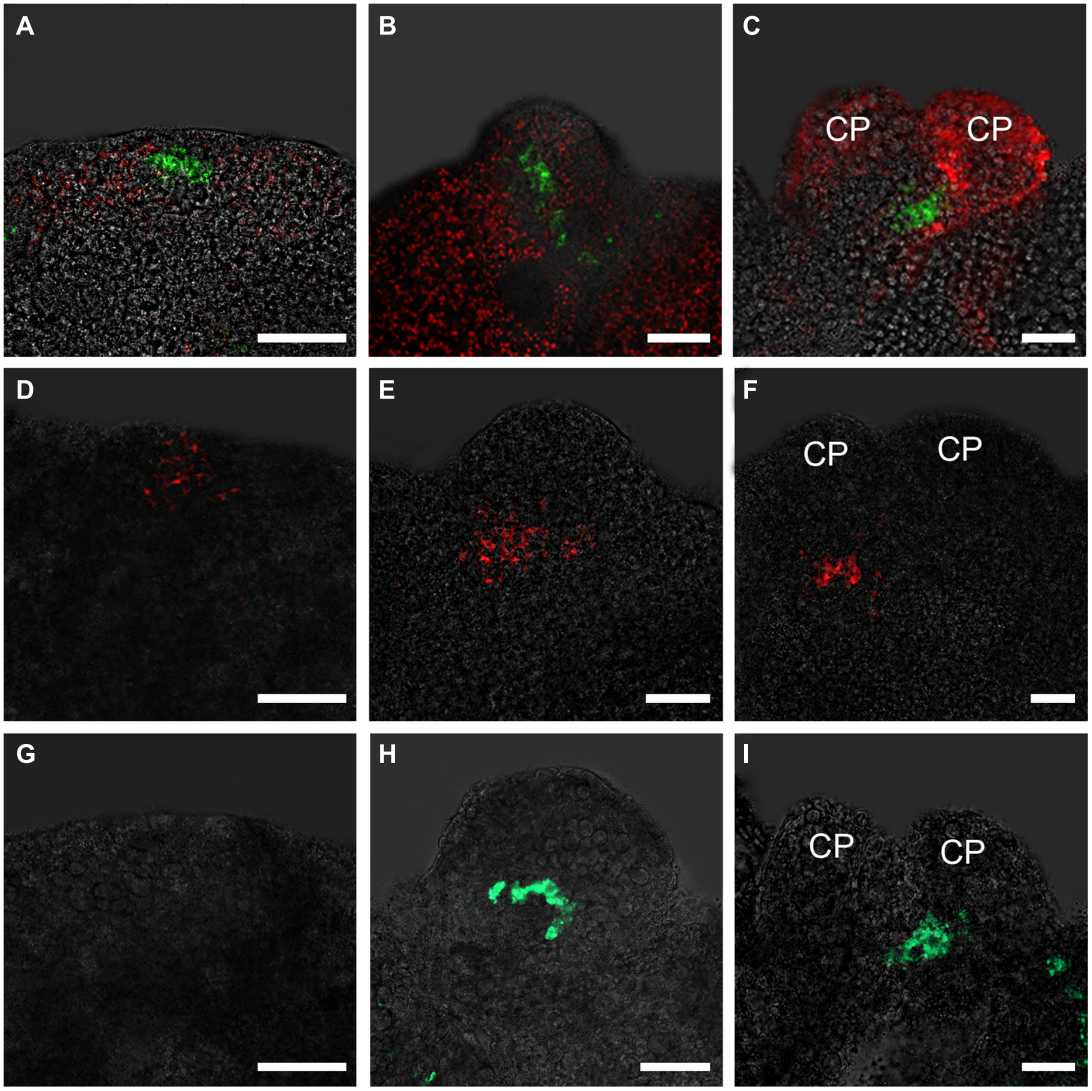
**Expression patterns of *WOX5*, *PLT2,* and *SCR* genes in embryonic calli during somatic embryogenesis. (A–C)** Expression patterns of *WOX5* indicated by *pWOX5::GFP* in embryonic calli induced in SEIM for 24 h (**A**; 83.72%, *n* = 86), 2 days (**B**; 84.27%, *n* = 89) and 3 days (**C**; 80.21%, *n* = 96). **(D–F)** Expression patterns of *PLT2* indicated by *pPLT2::RFP* in embryonic calli induced in somatic embryo-inducing medium (SEIM) for 24 h (**D**; 87.65%, *n* = 81), 2 days (**E**; 84.95%, *n* = 93) and 3 days (**F**; 90.53%, *n* = 95). **(G–I)** Expression patterns of *SCR* indicated by *pSCR::GFP* in embryonic calli induced in SEIM for 24 h (**G**; 87.36%, *n* = 87), 2 days (**H**; 89.58%, *n* = 96) and 3 days (**I**; 86.90%, *n* = 84). CP, cotyledon primordia. Red signals in **A–C** represent chlorophyll autofluorescence. Scale bars = 80 μm.

To analyze the spatiotemporally regulated formation of the root stem cell niche at the early stages of somatic embryogenesis, we examined the expression patterns of genes that play critical roles in root stem cell specification. Weak *pWOX5::GFP* signals were detected in a few internal regions but not in the edge regions of embryonic callus grown in ECIM for 14 days (data not shown). In contrast, after the embryonic calli were transferred to SEIM, stronger GFP signals started to be detected in some small edge regions at around 24 h (**Figure 1A**). At this time, the pro-embryos were not identifiable morphologically, but the callus cells with *WOX5* activity might have been the initial QC. Later, GFP signals were observed in the basal regions of the globular pro-embryos and then pro-embryos with cotyledon primordia (CP; **Figures 1B,C**). *PLT2* exhibited similar spatial expression patterns to *WOX5* at these stages (**Figures 1D–F**). Different from *WOX5* expression, *PLT2* expression was observed in a relatively large group of cells within the callus, which represented the root stem cell niche of the SE. We also determined the expression patterns of *SCR*, whose expression defines the position of the QC (**Figures 1G–I**). The GFP signals of *SCR* were first detected at 36–48 h after induction in SEIM, which was later than *WOX5* and *PLT2* expression. The expression patterns of genes for the QC and stem cell formation indicated that the RAM is established during SE induction.

### THE EMBRYONIC SHOOT–ROOT AXIS OF THE SE IS ESTABLISHED AT EARLY SOMATIC EMBRYOGENESIS

**FIGURE 2:**
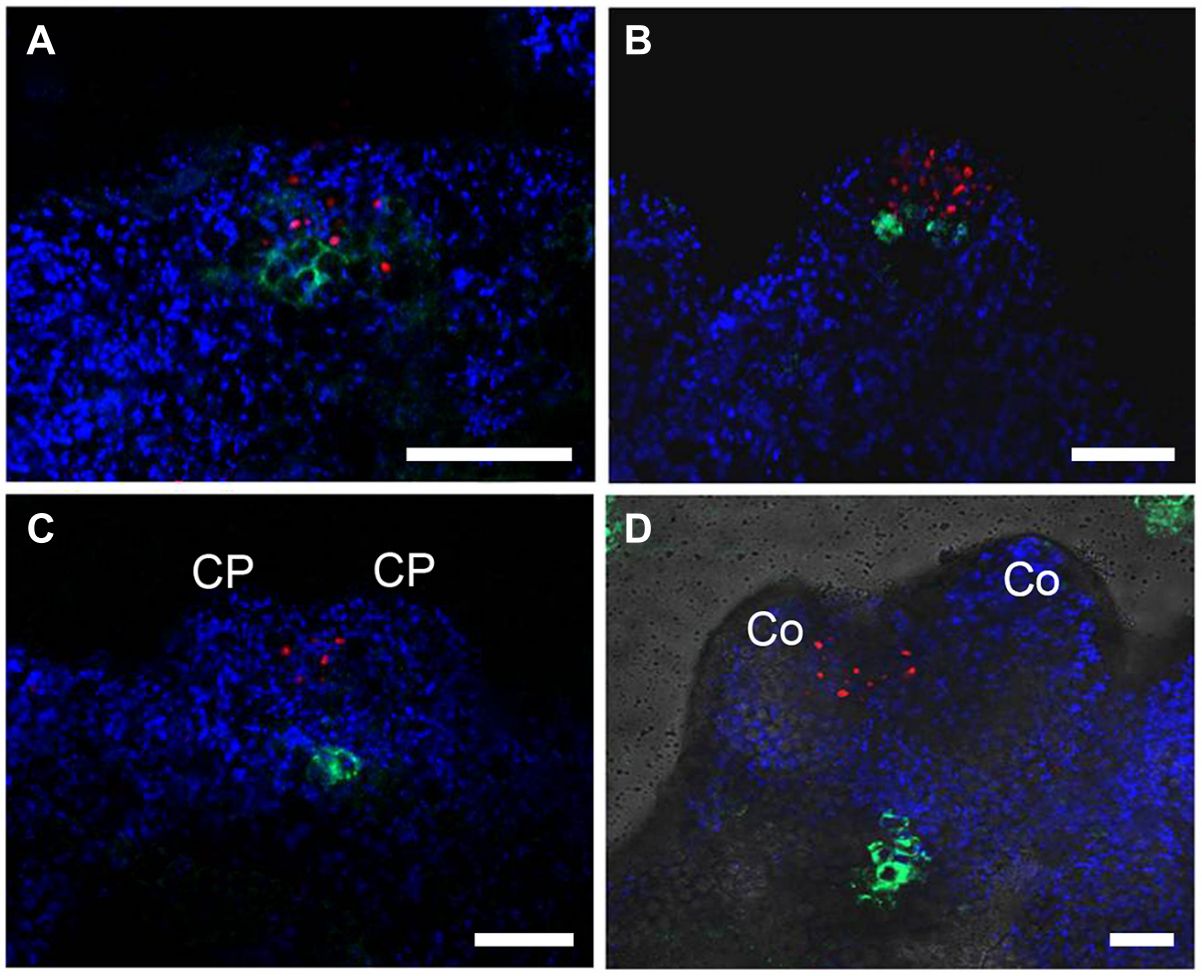
**Relative expression domains of *WOX5* and *WUS* genes.** *pWOX5::GFP* (green) and *pWUS::DsRed-N7* (red) signals in embryonic calli induced in SEIM for 24 h (**A**; 86.81%, *n* = 91), 2 days (**B**; 85.39%, *n* = 89), 3 days (**C**; 89.66%, *n* = 87) and 4 days (**D**; 87.37%, *n* = 95); CP, cotyledon primordia; Co, cotyledons. Blue signals represent chlorophyll autofluorescence. Scale bars = 80 μm.

To determine the relative expression domains of *WUS* and *WOX5*, we analyzed their co-localization using a *pWUS::DsRED-N7 pWOX5::GFP* marker line. *WUS* and *WOX5* transcription signals were first detected nearly overlapped at the edge regions of callus grown in SEIM for around 24 h (**Figure 2A**). At 36 h, the *WOX5* expression domain was just below and adjacent to that of *WUS* (**Figure 2B**). Subsequently, *WUS* transcripts were localized at the top regions between the CP of the pro-embryo, whereas *WOX5* transcripts were localized at the basal regions (**Figures 2C,D**). Thus, the SAM and RAM were initiated early and nearly overlapped in the edge regions of the callus, indicating that the apical–basal polarity of SE is determined and an embryonic shoot–root axis is established at the early stages of somatic embryogenesis.

### RAM-SPECIFIC *WOX5* AND *PLT* EXPRESSION IS REQUIRED FOR EMBRYONIC ROOT FORMATION AND SE INDUCTION

**FIGURE 3:**
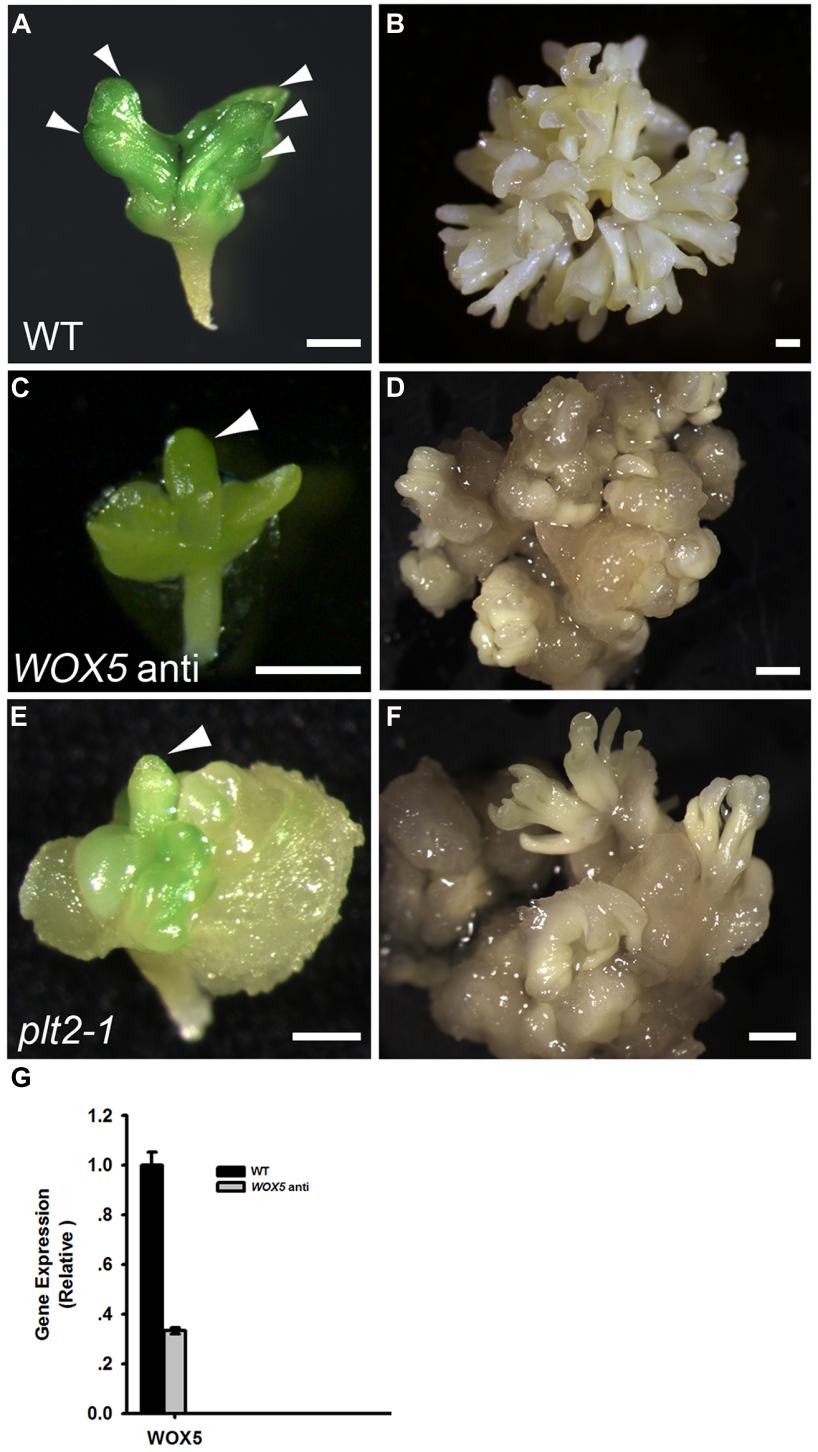
**Functional analysis of both *WOX5* and *PLT2* during somatic embryogenesis. (A,C,E)** Phenotypes of primary somatic embryos (PSE) induction from wild type (WT; **A**), *WOX5* antisense **(C)** and *plt2-1* mutant **(E)** explants. Arrowheads indicate the PSE. **(B,D,F)** Phenotypes of SSE induction from WT **(B)**, *WOX5* antisense **(D)** and *plt2-1* mutant **(F)** calli grown on SEIM for 8 days. **(G)** Expression levels of *WOX5* in estradiol-induced 15 days shoots of WT and *WOX5* antisense plants. Scale bars = 0.5 mm **(A,C,E)** and 1.2 mm **(B,D,F)**.

To determine the roles of WOX5 during somatic embryogenesis, we constructed a vector carrying antisense *WOX5* driven by an estradiol receptor-based transactivator, XVE (Zuo et al., 2000), and transferred it into plants. To monitor estradiol-induced production of cDNA-encoded transcripts, quantitative real-time PCR (qRT-PCR) was performed to detect expression levels of *WOX5* in 15 days shoots of wild type (WT) and *WOX5* antisense plants (**Figure 3G**). Estradiol was added every 2 days in medium. Immature zygotic embryos of the transgenic plants were used as explants. Green PSEs were induced on the shoot meristems of 84.47% of the explants after 10 days of culture on B5 agar medium containing 4.5 μM 2,4-D in light, without estradiol in the medium (**Figure 3A**; **Table 1**). After the PSEs were transferred from ECIM to SEIM, 62.26% of untreated calli produced SSEs and each embryonic callus generated 52.6 ± 7.6 normal SSEs (**Figure 3B**; **Table 1**). However, most explants carrying the antisense *WOX5* construct produced abnormal PSEs with deficient hypocotyl elongation and embryonic root formation in the presence of estradiol (**Figure 3C**; **Table 1**). Only 8.61% of embryonic calli carrying the antisense *WOX5* construct produced SSEs, and each embryonic callus generated only 3.3 ± 2.2 normal SSEs (**Figure 3D**; **Table 1**). We also examined PLT2′s function during SE induction. The *plt2-1* mutants generated PSEs with abnormal hypocotyls and embryonic roots (**Figure 3E**), as described for *plt1-1 plt2-1* double mutants (Su and Zhang, 2014). Embryonic calli of *plt2-1* mutants produced severely abnormal SSEs, without cotyledons or SAMs (**Figure 3F**; **Table 1**). Likewise, no hypocotyl- or root-like structures were observed on these SSEs. These results suggested that both WOX5 and PLT2 are required for SE formation.

**Table 1:** Somatic embryo (SE) regeneration frequencies of different mutants and transgenetic lines.

Mutant	*Wild type*	*WOX5* anti	*plt2-1*	35S::ARR7	35S::ARR15	*ahk2 ahk4*	*ahk3 ahk4*
Ratio^b^	84.47%	18.79%	25.56%	34.14%	26.67%	32.23%	30.54%
Ratio^c^	62.26%	8.61%	23.50%	21.05%	17.70%	15.95%	15.15%
Number^d^	52.6 ± 7.6	3.3 ± 2.2	17.2 ± 7.3	15.3 ± 5.6	13.5 ± 7.1	15.5 ± 7.6	12.2 ± 4.3

*^a^Mutant name; ^b^Proportion of explants that produced normal PSEs; ^c^Proportion of embryonic calli that produced normal SEs following culture in SEIM for 8 days; ^d^Number of normal SEs produced per embryonic callus following culture in SEIM for 8 days (mean ± SD, *n* ≥ 90).*

### SPATIOTEMPORAL DISTRIBUTION OF AUXIN RESPONSES IN EARLY SE INDUCTION

**FIGURE 4:**
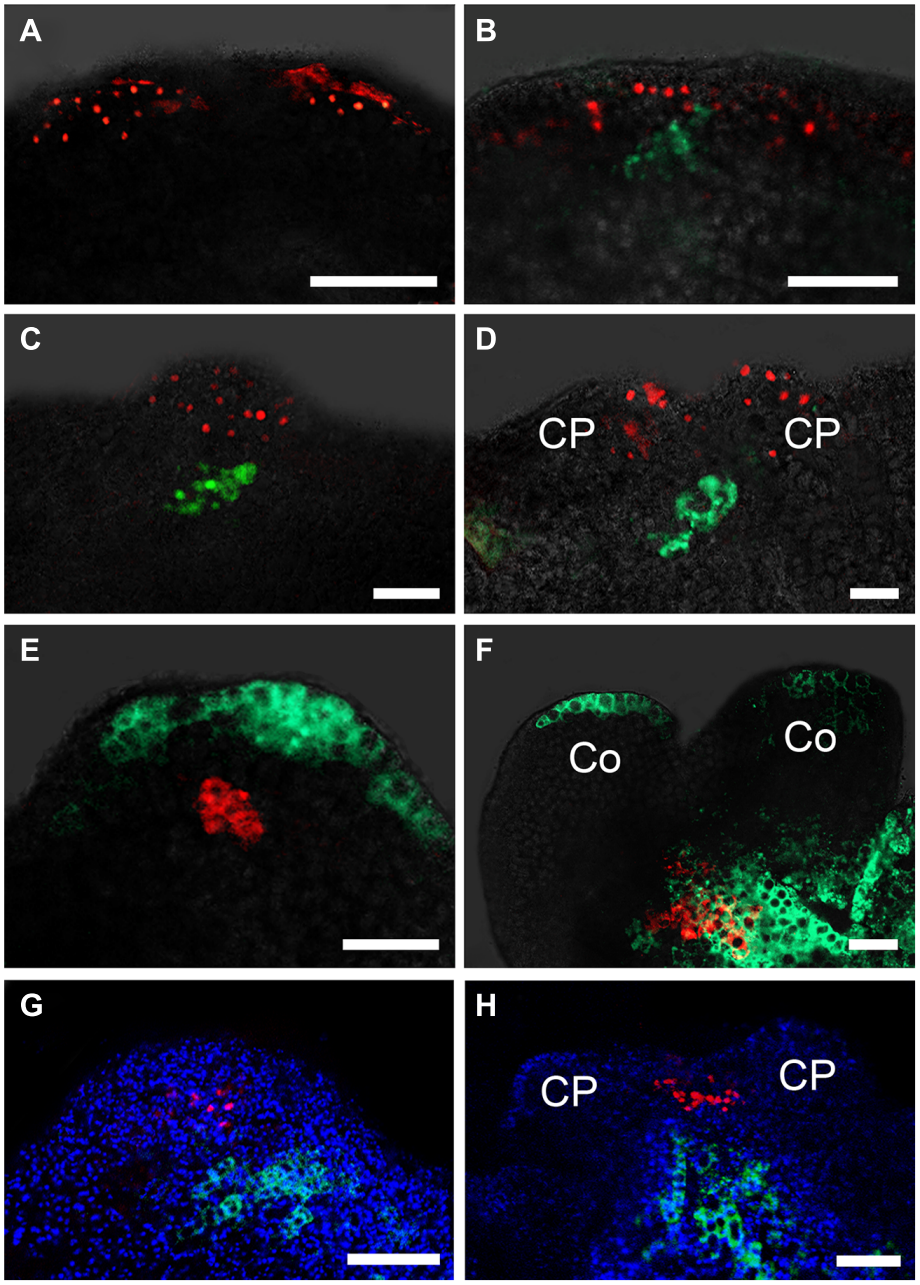
**Auxin and cytokinin responses in early somatic embryogenesis. (A–D)** Auxin response represented by *DR5rev:3XVENUS-N7* correlated with *WOX5* induction represented by *pWOX5::GFP* in embryonic calli induced in SEIM for 16 h (**A**; 88.24%, *n* = 85), 24 h (**B**; 83.33%, *n* = 96), 2 days (**C**; 87.50%, *n* = 88) and 3 days (**D**; 89.66%, *n* = 87). *pWOX5::GFP* signals are in green, *DR5rev:3XVENUS-N7* fluorescence signals are in red. **(E,F)** Auxin response represented by *DR5rev::GFP* correlated with *PLT2* induction represented by *pPLT2::RFP* in embryonic calli induced in SEIM for 3 days (**E**; 86.73%, *n* = 98) and 4 days (**F**; 90.91%, *n* = 77). *DR5rev::GFP* fluorescence signals are in green, *pPLT2::RFP* signals are in red. **(G,H)** Cytokinin response represented by *pARR7::GFP* correlated with *WUS* induction represented by *pWUS::DsRed-N7* in embryonic calli induced in SEIM for 2 days (**G**; 90.43%, *n* = 94) and 3 days (**H**; 85.37%, *n* = 82). *pARR7::GFP* fluorescence signals are in green, *pWUS::DsRed-N7* signals are in red, and chlorophyll autofluorescence is shown in blue. CP, cotyledon primordia; Co, cotyledons. Scale bars = 80 μm.

Previously, we reported that auxin response gradients were established in specific regions of the embryonic callus, and were responsible for SE formation (Su et al., 2009). Furthermore, we showed that the spatiotemporal distribution of the auxin response was correlated with the induced *WUS* expression at early somatic embryogenesis. Interestingly, we observed hardly any auxin response signals in the basal part of the somatic pro-embryo. We analyzed the auxin response signals and *WOX5* expression within the callus by double labeling with *DR5rev:3XVENUS-N7* and *pWOX5::GFP*. After 16 h incubation in SEIM, auxin response signals were detectable at the edge regions of the callus, but no *WOX5* signal could be detected (**Figure 4A**). At 24 h after induction, *WOX5* signals were detected in the region just beneath the outermost cell layers, where auxin response signals were identified (**Figure 4B**). After 48 h incubation, strong auxin response signals were detected at the upper part of the pro-embryo, but *WOX5* signals were localized at the basal part (**Figure 4C**). Later, auxin signals were redistributed to the top regions of the CP, and *WOX5* was continuously expressed in the basal part of the pro-embryo (**Figure 4D**). We also examined the auxin response in relation to *PLT2* expression through double labeling with *DR5rev:GFP* and the *PLT2* reporter *pPLT2:RFP*. Until 4 days after induction in SEIM, there were auxin signals distributed at the basal region where *PLT2* was expressed (**Figures 4E,F**). These results suggested that auxin response gradients were established in the SAM but not in the RAM of the pro-embryo during the early stages of somatic embryogenesis.

### CYTOKININ RESPONSES ARE SPATIOTEMPORALLY CORRELATED WITH RAM FORMATION

**FIGURE 5:**
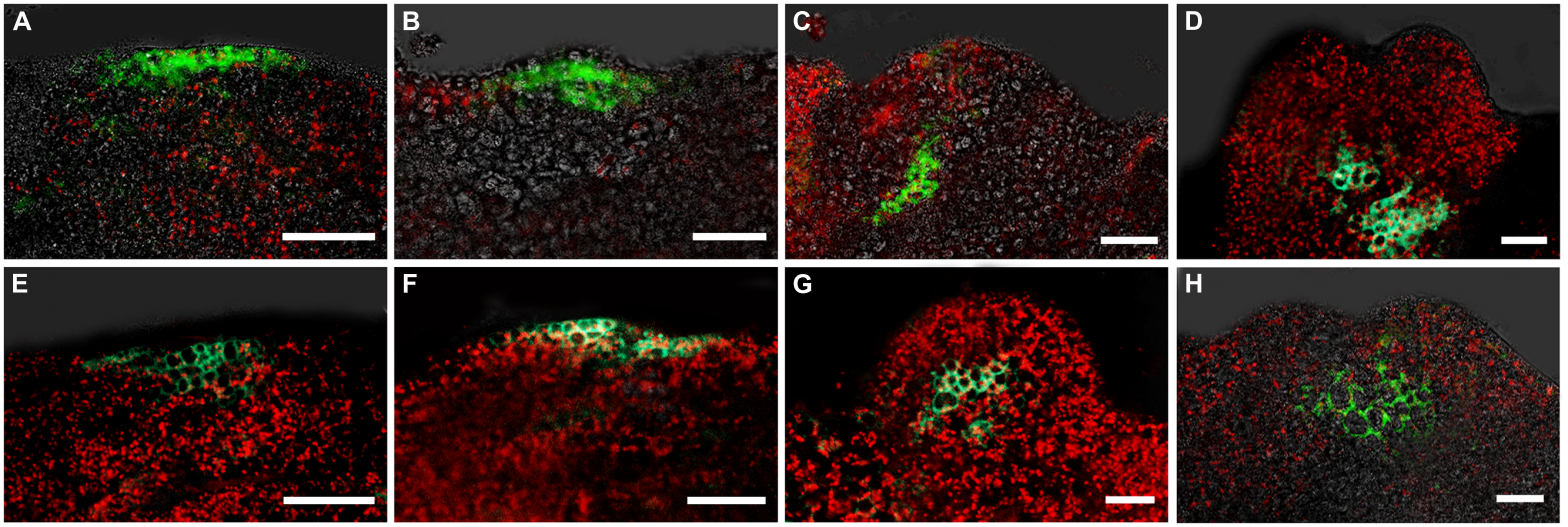
**Expression patterns of *ARR7* and *ARR15* during early somatic embryogenesis. (A–D)** Expression patterns of *ARR7* indicated by *pARR7::GFP* in embryonic calli induced in SEIM for 16 h (** A**; 93.02%, *n* = 86), 24 h (**B**; 90.53%, *n* = 95), 2 days (**C**; 85.88%, *n* = 85) and 3 days (**D**; 87.63%, *n* = 97). **(E–H)** Expression patterns of *ARR15* indicated by *pARR15::GFP* in embryonic calli induced in SEIM for 16 h (**E**; 88.24%, *n* = 85), 24 h (**F**; 82.29%, *n* = 96), 2 days (**G**; 94.05%, *n* = 84) and 3 days (**H**; 86.96%, *n* = 92). Red signals represent chlorophyll autofluorescence. Scale bars = 80 μm.

It has been reported that both auxin and cytokinin responses are critical for specifying the root stem cell niche in embryos (Müller and Sheen, 2008). To determine how the cytokinin response occurs in callus when SEs are induced, we analyzed the spatiotemporal expression patterns of *ARR7* and *ARR15*, which are primary responsive genes in cytokinin signaling and can be rapidly induced by cytokinin (To and Kieber, 2008; Werner and Schmülling, 2009). Signals of *pARR7::GFP* were first detected at some small regions of the calli near the edge at 24 and 36 h after induction in SEIM (**Figures 5A,B**), which was similar to the auxin response at these stages. Interestingly, after 2 days induction in SEIM, the signals were restricted to the basal part of the pro-embryo rather than the top (**Figures 5C,D**). We also used the *pARR15::GFP* reporter to examine the expression patterns of *ARR15*, and found similar distribution patterns of GFP signals to those of *ARR7* (**Figures 5E–H**). These results showed that the cytokinin response occurs in the regions of SE initiation, but the cytokinin response patterns are different from those of the auxin response. To examine whether the cytokinin response is correlated with the establishment of the embryonic SAM, we visualized the cytokinin response using a *pARR7::GFP pWUS::DsRED-N7* marker line. The distribution regions of cytokinin signaling were quite different from those of *WUS* expression, which was localized in the opposite pole of the pro-embryos (**Figures 4G,H**). We further examined the cytokinin response in relation to *WOX5* expression through double labeling with the *pARR7::GFP* and *PWOX5::RFP* reporters (Su and Zhang, 2014). Strong cytokinin responses were induced in the restrictive regions substantially overlapping with the *WOX5* signals. Thus, the results suggest that establishment of the cytokinin response is correlated not with *WUS* but with *WOX5* induction within the callus, implying that cytokinin is required for embryonic RAM initiation.

### CYTOKININ SIGNALING IS REQUIRED FOR EMBRYONIC RAM REGENERATION AND SE INDUCTION

**FIGURE 6:**
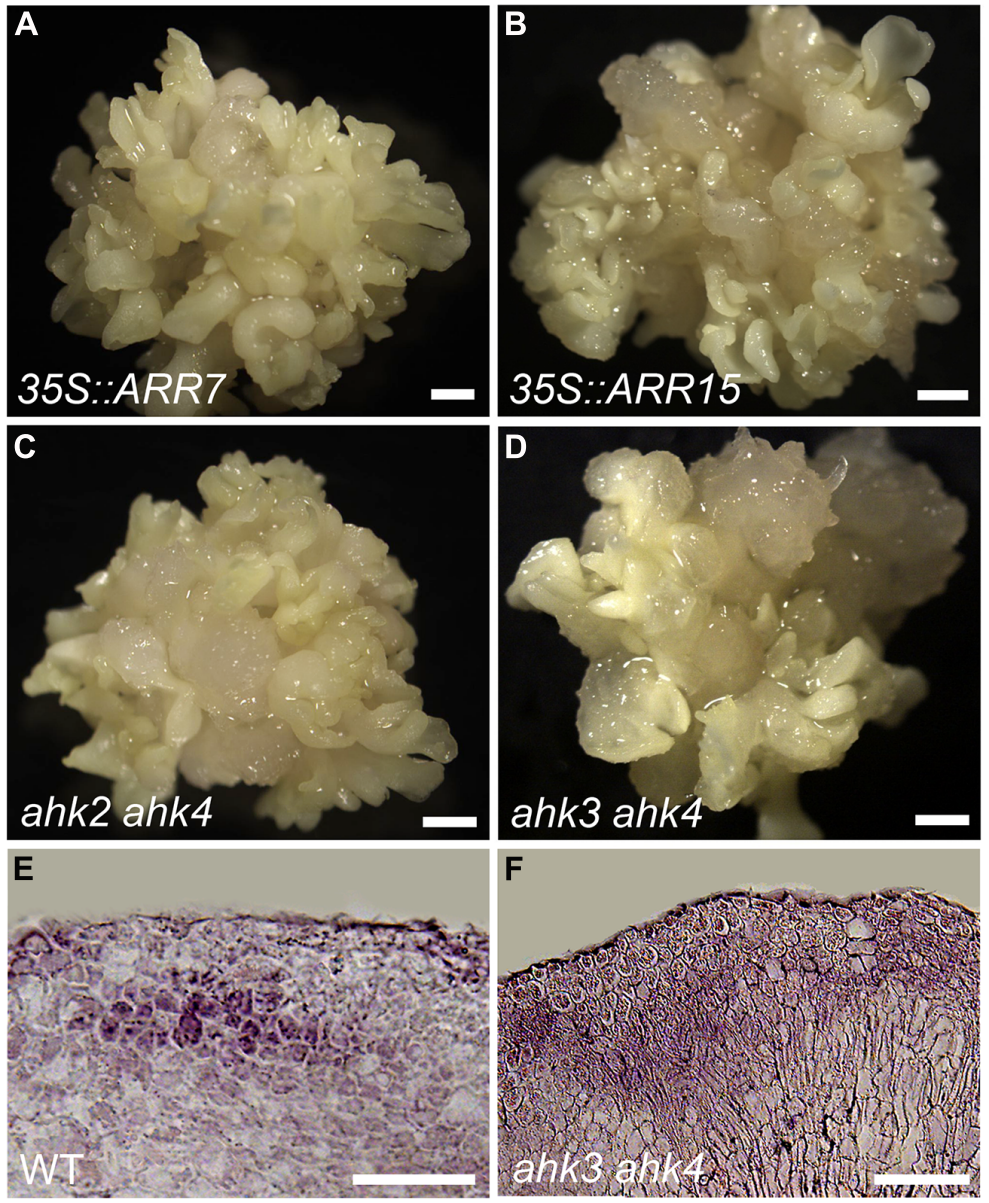
**Functions of cytokinin signaling on SE induction. (A–D)** Phenotypes of SSE induction from 35S::ARR7 **(A)**, 35S::ARR15 **(B)**, *ahk2 ahk4* mutant **(C)**, and *ahk3 ahk4* mutant **(D)** calli grown on SEIM for 8 days.** (E)** *WOX5* transcript signals in WT embryonic callus cultured in SEIM for 24 h; 86.15%, *n* = 35. **(F)** Dispersed *WOX5* transcript signals in *ahk3 ahk4* double mutant callus following culture in SEIM for 24 h; 87.30%, *n* = 33. Scale bars = 1.2 mm **(A–D)** and 80 μm **(E,F)**.

The spatial distribution of cytokinin responses detected through *ARR7* and *ARR15* transcriptional signal profiles prompted us to confirm whether a functional cytokinin signaling mechanism is necessary for normal somatic embryogenesis. *ARR7* and *ARR15* act as negative regulators of cytokinin signaling by repressing type-B *ARRs* via unknown mechanisms (To and Kieber, 2008; Werner and Schmülling, 2009). Overexpression of *ARR7* and *ARR15* can attenuate cytokinin signaling to a sufficiently low level, resulting in reduced sensitivity to cytokinin in root elongation and shoot formation or an early flowering phenotype (Werner and Schmülling, 2009). To facilitate functional analysis of cytokinin signaling, we generated transgenic plants overexpressing *ARR7* or *ARR15* under the control of the CaMV 35S promoter. The *ARR7*-overexpressing explants showed a severely defective PSE phenotype without normal elongated hypocotyls or obvious embryonic roots (Su and Zhang, 2014). Subsequently, 78.95% of PSEs generated severely abnormal SSEs after induction in SEIM (**Figure 6A**; **Table 1**). Similar to *ARR7*-overexpressing plants, *ARR15*-overexpressing plants also generated abnormal PSEs with defective hypocotyls and embryonic roots, and subsequently, abnormal SSEs (Su and Zhang, 2014; **Figure 6B**). In *Arabidopsis*, three histidine kinases (AHKs), AHK2, AHK3, and AHK4, positively regulate cytokinin-signaling as direct receptors of cytokinin (To and Kieber, 2008). Thus, we further analyzed the developmental characteristics of *ahk2 ahk4* and *ahk3 ahk4* double mutant calli during SE regeneration. The phenotypes of both double mutants were consistent with previous descriptions of *ARR7*- and *ARR15*-overexpressing plants (**Figures 6C,D**; **Table 1**). Interestingly, we found that SE regeneration was impaired with defective cytokinin signaling, which was similar to *WOX5*-antisense plants and *plt2-1* mutants.

Because the expression patterns of *WOX5* were quite similar to the cytokinin response distribution, we hypothesized that cytokinin signaling might regulate *WOX5* expression for early SE induction. We performed *in situ* hybridization to analyze *WOX5* expression in the *ahk3 ahk4* double mutant. Indeed, the *WOX5* expression pattern was greatly disrupted in the *ahk3 ahk4* double mutant compared with the WT after the callus was transferred into SEIM (**Figures 6E,F**). *WOX5* signals were restricted to the site of the future embryonic root meristem in the WT (**Figure 6E**), whereas the localization of *WOX5* signals was stronger and more dispersed in the *ahk3 ahk4* mutant (**Figure 6F**). These results indicated that cytokinin signaling negatively regulates *WOX5* expression in the proper pattern for initiation of the embryonic RAM.

## DISCUSSION

During somatic embryogenesis, the developmental process from the globular stage to the torpedo stage shares considerable similarity with that of zygotic embryogenesis (Meinke, 1991; Zimmerman, 1993). Although there are many similarities in the morphological and cellular programs of both zygotic and somatic embryogenesis, the mechanisms determining the initiation of these two processes might be different. The specific characteristics of somatic embryogenesis could be due to the origination of the somatic embryo from embryonic callus, not a zygote as in zygotic embryogenesis.

### THE ORIGINS OF EMBRYONIC SAM AND RAM WERE QUITE DIFFERENT BETWEEN SOMATIC EMBRYOS AND ZYGOTIC EMBRYOS

In *Arabidopsis*, the mature embryo displays a main shoot–root axis of polarity, with the correct relative positioning of the embryonic SAM at the top and RAM at the opposite pole, separated by the hypocotyl (embryonic stem; Jürgens, 2001; Friml et al., 2003). The origin of this apical–basal pattern of shoot–root axis has been traced back to early embryogenesis, when zygotic division generates a smaller apical and a larger basal cell. After the apical domain of the pro-embryo has been specified, the embryonic SAM is initiated by the onset of *WUS* expression in the four subepidermal apical cells of the 16-cell embryo (Mayer et al., 1998; Laux et al., 2004). Subsequently, the QC of the RAM is established at approximately late globular embryo stage and marked by the expression of *WOX5* (Wysocka-Diller et al., 2000; Haecker et al., 2004).

During somatic embryogenesis, *WUS* and *WOX5* were simultaneously activated in nearly overlapped callus cells, when somatic pro-embryos could not be identified morphologically (**Figure 2**; Su et al., 2009). The nearly overlapped spatial relationship between regenerated embryonic SAM and RAM during SE initiation represents the different origins of apical–basal pattern between somatic embryos and zygotic embryos. It is likely that SEs initiate from specific embryonic callus cells which acquire features similar to meristematic cells. These specific embryonic cells of callus are reprogrammed and determined to form cells of both OC and QC for embryonic SAM and RAM formation. In addition, early defects in RAM initiation with inhibited *WOX5* expression also affected the initiation of the SAM, probably by disrupting the apical–basal pattern of early somatic embryogenesis. These results suggest that QC signaling not only maintains stem cell identity in the RAM but also is crucial for OC cells initiation, implying that the stem cell niches of the RAM and the SAM share developmental correlations during SE initiation.

### CYTOKININ RESPONSE WAS INVOLVED IN INDUCING CORRECT *WOX5* EXPRESSION AND RAM FORMATION

The patterns of embryonic SAM and RAM establishment in SE initiation suggest the presence of inductive hormonal signals to position them within the embryonic callus. Given the positive effects of auxin on *WUS* expression and SE induction (Su et al., 2009; **Figure 4**), it is likely a candidate factor that is required for embryonic RAM formation. Here, we found a cytokinin response distribution established in the regions where *WOX5* and *PLT2* were initiated (**Figures 1** and **5**). RAM formation and SE regeneration were severely inhibited in transgenic plants overexpressing *ARR7* or *ARR15* and in the *ahk* mutants, in which cytokinin signaling was inhibited (**Figure 6**). Moreover, in cultured tissues of the *ahk* mutants, *WOX5* expression patterns were seriously disturbed compared with control tissues (**Figure 6**). Thus, we hypothesize that removal of exogenous auxin may be a stress factor that causes cytokinin polar distribution and responses in specific regions, which induce correct *WOX5* expression and subsequent SE initiation. Induced *WOX5* transcripts were continuously detectable in areas of high cytokinin response (Su and Zhang, 2014), suggesting that cytokinin functions in the initiation and maintenance of the embryonic RAM during somatic embryogenesis. The positive action of cytokinin in SAM regeneration has been reported in several studies (Pernisová et al., 2009; Buechel et al., 2010). Treatment with high levels of exogenous cytokinin induces cell proliferation and stimulates shoot regeneration (Skoog and Miller, 1957). Cytokinin induces *WUS* expression during *in vitro* establishment of the SAM from cultured root explants (Gordon et al., 2009). A cytokinin response occurs in the center of the regenerated SAM, overlapping with *WUS* expression regions (Cheng et al., 2013). In contrast, an opposite effect of cytokinin in root regeneration has been observed. Cytokinin influences auxin-induced RAM regeneration via regulation of PIN-mediated auxin polar transport (Pernisová et al., 2009). Therefore, the functions of cytokinin in RAM establishment during SE initiation differ from those in shoot and root regeneration.

### SPATIOTEMPORAL DISTRIBUTION OF AUXIN AND CYTOKININ RESPONSE IN EMBRYONIC CALLUS DETERMINES ESTABLISHMENT OF SE SHOOT–ROOT AXIS

Cytokinin and auxin appear to be the most important hormones in the regulation of organ regeneration (Moubayidin et al., 2009; Su et al., 2011). A high exogenous auxin/cytokinin ratio induces root regeneration, whereas a low ratio promotes shoot induction (Skoog and Miller, 1957). Recent studies have suggested that exogenous hormones treatment is the critical factor triggering biosynthesis and response of endogenous hormones in early developmental events of *in vitro* regeneration. Specialized endogenous hormonal signaling is required for specific cell differentiation that determines the developmental fate of callus cells (Gordon et al., 2007; Su et al., 2009, 2011). During early somatic embryogenesis, removal of exogenous auxin triggers the regional distribution of endogenous auxin response in callus surrounding areas of *WUS* expression initiation (Su et al., 2009). Following *WUS* induction, distribution of auxin response was re-established in the SAM region. In contrast, the distribution of cytokinin-response signal in callus overlapped with the areas of *WOX5* expression (Su and Zhang, 2014). These results imply that establishment of auxin and cytokinin response patterns within callus plays an important role in *WUS* and *WOX5* regional expression and shoot–root axis formation. Furthermore, auxin response signals accumulated at the basal region of pro-embryos following prolonged incubation in SEIM (**Figures 4E,F**). The redistributed auxin response corresponded to *PLT2* expression at the later stages of SE development. Therefore, our results suggest that cytokinin and auxin are key players in axial patterning of the SE, especially in shoot and root meristem initiation. The mechanisms of hormonal regulation in SE initiation are quite different from those in shoot or root regeneration individually, which remains a major challenge for the future.

## AUTHOR CONTRIBUTIONS

Ying Hua Su and Xian Sheng Zhang designed the research. Ying Hua Su and Yu Bo Liu performed the research. Bo Bai analyzed the data. Ying Hua Su and Xian Sheng Zhang wrote the paper.

## Conflict of Interest Statement

The authors declare that the research was conducted in the absence of any commercial or financial relationships that could be construed as a potential conflict of interest.
